# Psychosocial, behavioural and health system barriers to delivery and uptake of intermittent preventive treatment of malaria in pregnancy in Tanzania – viewpoints of service providers in Mkuranga and Mufindi districts

**DOI:** 10.1186/1472-6963-14-15

**Published:** 2014-01-13

**Authors:** Godfrey M Mubyazi, Paul Bloch

**Affiliations:** 1 National Institute for Medical Research (NIMR), Department of Health Systems and Policy Research, Centre for Enhancement of Effective Malaria Interventions (CEEMI), 2448 Barak Obama Drive (former Luthuli/Ocean Road), P.O Box 9653, Dar es Salaam, Tanzania; 2 Steno Diabetes Center, Steno Health Promotion Center, Gentofte, Denmark

## Abstract

**Background:**

Intermittent preventive treatment of malaria in pregnancy (IPTp) using sulphurdoxine-pyrimethamine (SP) is one of key malaria control strategies in Africa. Yet, IPTp coverage rates across Africa are still low due to several demand and supply constraints. Many countries implement the IPTp-SP strategy at antenatal care (ANC) clinics. This paper reports from a study on the knowledge and experience of health workers (HWs) at ANC clinics regarding psychosocial, behavioural and health system barriers to IPTp-SP delivery and uptake in Tanzania.

**Methods:**

Data were collected through questionnaire-based interviews with 78 HWs at 28 ANC clinics supplemented with informal discussions with current and recent ANC users in Mkuranga and Mufindi districts. Qualitative data were analysed using a qualitative content analysis approach. Quantitative data derived from interviews with HWs were analysed using non-parametric statistical analysis.

**Results:**

The majority of interviewed HWs were aware of the IPTp-SP strategy’s existence and of the recommended one month spacing of administration of SP doses. Some HWs were unsure of that it is not recommended to administer IPTp-SP and ferrous/folic acid concurrently. Others were administering three doses of SP per client following instruction from a non-governmental agency while believing that this was in conflict with national guidelines. About half of HWs did not find it appropriate for the government to recommend private ANC providers to provide IPTp-SP free of charge since doing so forces private providers to recover the costs elsewhere. HWs noted that pregnant women often register at clinics late and some do not comply with the regularity of appointments for revisits, hence miss IPTp and other ANC services. HWs also noted some amplified rumours among clients regarding health risks and treatment failures of SP used during pregnancy, and together with clients’ disappointment with waiting times and the sharing of cups at ANC clinics for SP, limit the uptake of IPTp-doses.

**Conclusion:**

HWs still question SP’s treatment advantages and are confused about policy ambiguity on the recommended number of IPTp-SP doses and other IPTp-SP related guidelines. IPTp-SP uptake is further constrained by pregnant women’s perceived health risks of taking SP and of poor service quality.

## Background

Malaria is an infectious disease transmitted by specific mosquitoes, a public health problem facing many tropical and sub-tropical countries, and associated with substantial risks to the mother, foetus and newborn [1,2]. National, bilateral and multilateral organizations as well as other development stakeholders/partners have been working hard toward achieving a significant reduction of the occurrence and burden of this disease. Despite many health programmes having been instituted and others still being recommended, besides the advances in the newly recommended control methods, malaria in pregnancy (MiP) and in children under five years remains one of topmost public health problems calling for attention to be paid to in tropical and sub-tropical countries. This is due to the persisting epidemiological, systemic and operational challenges, and tropical sub-Saharan Africa (SSA) is the region mainly hit [2,3]. That is why the World Health Organization (WHO) has urged ministries of health (MoH) in malaria endemic countries to ensure that they prioritize identification, institutionalizing and strengthening of all measures aimed at promoting effective control of MiP and in under-five children who are most vulnerable to malaria infections and their morbidity and mortality consequences [2,4].

One of the advocated approaches for effective control of diseases in general is to ensure that basic preventive services as well as curative services are in place in the health care system [5]. Where possible, attempt to achieve universal coverage by offering the basic (primary) health care services free of charge to all people in need irrespective of their incomes is highly recommended. However, there has been a prolonged research and policy debate on whether this policy ambition is realistic and, if so, would lead to the anticipated universal service coverage within and outside Africa [6-8].

Intermittent preventive treatment of malaria during pregnancy (IPTp) using sulphadoxine-pyrimethamine (SP) is a strategy for malaria control in pregnant women that became officially recommended by the WHO in 2000. Since then, this strategy has become an integral package of national health care systems in SSA, particularly for protecting the targeted women and their pregnancies in areas with moderate to high malaria transmission. This strategy requires each pregnant woman in malaria endemic countries to receive a full therapeutic standard dose of SP at defined period, particularly allowing a one month period apart between one dose and the next one [2,4]. The IPTp guidelines recommend that the administration of the first dose (abbreviated as IPTp-1) should be done during the first trimester of pregnancy, but immediately after quickening while the second dose (IPTp-2) and, possibly, a third dose (IPTp-3) should be administered during the third trimester. That is to say that more than two doses are allowed for the women who manage to complete at least four scheduled ANC clinic visits. Meanwhile, it is suggested that pregnant women eligible for taking these doses have to be supervised by qualified health service providers under the directly observed therapy (DOT) procedure [5]. When the SP is administered together with other supplementary medicines such as ferrous/iron and folic acid tablets, it is recommended that care should be taken to follow the official guidelines. In fact, a dose of 30-60 mg of iron and 0.4 mg of folic acid should be given daily to supplement SP per pregnant woman to reduce the risk of low birth weight in infants, maternal anaemia, and iron deficiency term. Moreover, SP can be administered to a pregnant woman with an empty stomach (hungry clients) or who not hungry for IPTp, but where it is noted that the client concerned is presents symptoms of malaria and get confirmed to be parasitemic, then she should not receive IPTp and instead should be treated with the appropriate drug recommended in the existing WHO/National guidelines [5].

In Tanzania, MiP sustains high levels of morbidities and mortalities among pregnant women and newborn babies [9,10], and it accounts for at least 20% of all maternal deaths in some districts [11]. In this country, maternal deaths have remained as high as over 500 per 100,000 live births for almost the past two decades without remarkable decline [12]. Therefore, the latest records showing that maternal mortality ratio (MMR) caused by a combination of malaria, HIV/AIDS and other conditions has declined to 454 deaths per 100,000 live births, the infant mortality rate caused by malaria and other conditions being estimated at 51 per 1,000 population [13], and under five mortality rate attributable to malaria and anaemia being estimated to be 23% [12], are subject to further and review if possible debate. In Tanzania, the IPTp-SP strategy was put into operation in 2001 [13,14]. Initially, the Ministry of Health and Social Welfare (MoHSW)’s aimed to achive at least 60% coverage of all pregnant women with at least two doses of IPTp-SP by 2005 in line with the Abuja Declaration of 2000. However, the target was later on increased to a coverage rate of 85% pregnant women attending ANC clinics by 2010 [15]. The latter decision was in line with the Roll Back Malaria Partnership’s aim that emphasized countries to target covering at least 80% of all the pregnant women living in areas with stable or high intensity of malaria with IPTp doses. The same projection was made about targeting to cover pregnant women using ITNs by 2010. Discouragingly, most countries did not succeed attaining this target due to a number of demand and supply barriers [16].

The issue of administration of IPTp-SP following the existing national guidelines in Tanzania has remained precipitating debates. As reported before, at least a number of frontline HWs interviewed at various health care facilities (HFs) have criticised the ambiguities existing in the existing national guidelines in Tanzania. They pointed out the guidelines emphasizing the IPTp-1 dose to be administered in the 20-24 weeks of gestational age of pregnancy and IPTp-2 during the 28^th^ - 32^nd^ weeks gestational age. To them, this seemed contrary to WHO guidelines recommending IPTp-SP administration immediately after quickening and even at late stages of pregnancy including the time close to delivery [5]. The national guidelines to which reference was made by the reporting workers have for a long time guiding the service provider not to administer SP after the 36^th^ week of their pregnancy because of the anticipated risks [14]. From the perspective of other critics, this HW’s feeling cannot be counted as the failure of such individuals to comply with the requirements if they strictly adhered to giving SP not later than the stated period, and instead it is a reflection of a policy weakness in the first place [17].

IPTp implementation in Tanzania has a wider chance of being widely implemented at the HFs owned by both the private and public authorities. So, it is a matter of where the client(s) seeks the services and the availability of the institutional environments that are supportive for the services needed to be accessed and utilised. Records indicate that nearly all public and many private (for-profit and not-for-profit) HFs in Tanzania provide ANC services or have ANC clinics, and over half of the Tanzanian population live within 5km of a nearby HF. Meanwhile, critics argue that despite the official records indicating that over 80% of the pregnant women in Tanzania are noted of having physical access to ANC services, they may not all and always utilise the services available at the existing facilities due to various physical, financial and process barriers [18]. That is why it is important for the national malaria control program (NMCP) and officers to note that achieving higher ANC (including IPTp-SP) coverage rates is already constrained. They should recognize that high service coverage would depend on conditions related to the availability of essential medicines and other supplies, HWs’ and users’ compliance with guidelines as well as affordability of the services, and real or perceived quality of the services at the existing HFs [17,19]. The available evidence indicates that so far generally the proportion of pregnant women completing four ANC visits in Tanzania is low. Records reveal that the overall coverage is 43% for both urban and rural areas, while specifically it is 55% for urban areas only and only 39% for rural areas [20].

In a nutshell, technical research reports, journal-based publications and official working papers/documents indicate the numerous factors constraining the delivery and uptake of IPTp to include the following categories: (i) psychosocial and cultural; (ii) economic (iii) systemic [3,21,22]; (iv) biomedical; and (v) political. Issues that have received substantial attention include (a) how to provide optimal IPTp services to HIV positive clients [5,23]; (b) how to optimise, balance and combine advocacy and health communication on IPTp, insecticide-treated nets (ITNs) and indoor residual spraying (IRS) [24]; and (c) how to optimise administration of IPTp-SP in areas with low and moderate malaria transmission intensity [1]. Meanwhile, given the diverse nature of the challenges experienced at national and local levels, suggestions have been given that more studies are required to evaluate the status of IPTp implementation and provide recommendations for optimising the coverage and quality of services [21,25].

In Tanzania, documented psychosocial challenges are mainly related to negative social perceptions on SP’s safety and treatment benefits [25-27]. Economic challenges are related to the availability/supply of SP at HF level [3,17,18], how to provide IPTp-SP and ITNs free of charge to all eligible clients [28], and costs/affordability of SP to the clients using the private and public HFs [29,30].

Within the framework of the National Package of Essential Reproductive and Child Health (RCH) Interventions, the Ministry of Health and Social Welfare (MoHSW) of Tanzania launched the focused ANC (fANC) programme in 2004. This programme was revised in 2007 [12]. Frontline HWs were expected to use the fANC guidelines as reference material when administering IPTp-SP to pregnant women at ANC clinic levels. The fANC service package identifies a range of services which are essential for every ANC client. These include, among others, physical and clinical examination, laboratory screening for haemoglobin levels, HIV testing, syphilis testing, urine analysis, blood grouping and cross-matching, IPTp-SP through DOT, and administration of ferrous/folic acid tablets. Other elements include history taking, health education, health counseling (including counseling for HIV/AIDS), and basic vaccination/immunization. Furthermore, the frontline service providers are required to collect, keep and report on specific records of their clients related to delivered services and observed health status. This should be part of the essential national health information system to facilitate local and national decision-making including health service planning [15,31]. Since the fANC was introduced, its operational effectiveness (its real practicability potential) has not been systematically evaluated. That is why little information is so far available regarding the acceptability, accessibility, practicability and quality of fANC services [21,27]. As suggested earlier, a proper evaluation should have included insights or analysis and experience of frontline HWs who are responsible for implementing it [2], and this is important because the HWs may be challenged by inadequate supplies, logistics and manpower [32-34] on one hand and by socio-cultural, cost-related, and provider-client interaction factors on the other hand. All these factors either in singularity or in combination have a major lowering impact on the quality and uptake of services [27,35].

This paper, therefore, is relevant as it reports on a study that assessed the psychosocial, cultural, behavioural and systemic determinants of pregnant women’s attendance to ANC clinics and their ability to access and use IPTp-SP services. The data presented and analysis made on them help to narrow the current evidence gap and identify other potential areas needing further systematic research. Thus, the present paper presents the perspectives of HWs who have been dealing with ANC services directly as service providers or service managers at HF levels in two districts in Tanzania. It adds on the previous report from the same study districts on the equivalent perspectives of ANC users [18], partly the service providers [29], and of higher level stakeholders at district and national level [17,36,37]. These findings provide additional evidence in support of the reports from a previous survey in Korogwe district also in Tanzania [26] and the reviewed literature on previous studies in the same country and abroad [27].

## Methods

### Study design

The main study from which the findings of this paper were obtained was designed as a cross-sectional survey made in Mkuranga and Mufindi districts in Tanzania. The main objective in that survey was to assess the economic and other contextual determinants of acceptability and practicability of the malaria IPTp-SP strategy [38]. Among the target population groups were the frontline HWs who were traced at public and private HFs implementing ANC services. The perspectives of these respondents have been partly supported and partly challenged by the experiences obtained from the users of the existing ANC clinics/services, particularly pregnant women also assessed during the present study period as well as the lactating mothers who were recently using such services. Additional views were obtained from the national and district level officers [17,18].

### Study sites

Mkuranga is located in the Pwani (officially known as Coast) Region in the East near the Indian Ocean, an area of stable and perennial malaria transmission. Mufindi is located in Iringa Region in the southern highlands of Tanzania, an area of seasonal malaria transmission. Selection of these districts was done purposefully and based on criteria related to their locations in regions with considerable variations in malaria transmission intensity [39], socio-economic characteristics and HF profiles [18,29,36,37].

### Study population (HWs) and health facilities identified

#### Health facilities

In Mufindi, there was a total of 59 HFs in 2006 when the main data collection process took place. Of these HFs, 44 belonged to government (i.e. were public) and the rest were private as 10 were owned by faith-based organizations (FBOs), operating as private-not-for-profit entities while 5 were commercial (private-for-profit). Further categorization of HFs indicated that there were only 5 health centres (HCs) out of a total of the HFs. Four of such HCs were public, the remaining one namely *Usokami* belonged to the Roman Catholic Church – a faith-based organization (FBO). There were only 2 hospitals, namely Mafinga District Hospital located in the capital of Mufindi district – Mafinga, and this hospital is public (owned by the government). The second hospital was Lugoda Hospital but this belongs to a private Tea Company and was located some miles in the interior/remote setting far from Mafinga Town. There were 52 dispensaries among which 4 were commercial, 10 were owned by faith-based whereas the rest were public.

In Mkuranga, there were 29 HFs in total in 2006, and of these HFs, only 1 was a hospital namely Mkuranga District Hospital - belonging to the government and located in the capital of the district (Mkuranga Town), about 60km from the centre of the City of Dar es Salaam [29]. There were 4 HCs, but only 3 of these were functioning for many (>10) years and were all public. The remaining 1 HC was owned by the RC Church, but was still new and less popular to the majority of community members and ANC clients. There were 25 dispensaries in total throughout the district, among which 15 were public while the rest were private (and among these about half were commercial i.e. private for profit).

#### Health care workers (HWs)

In total, 78 HWs from both districts (35 from Mkuranga, 43 from Mufindi) were included in the study. These were identified at HFs owned by different authorities/organizations. In Mkuranga, the HWs were identified at 13 HFs while in Mufindi they were identified at 15 HFs. The HFs included those of public nature and the private ones (both commercial and faith-based). Three of the HFs from which the HWs were identified were hospitals (including 1 district hospital in Mkuranga and 2 hospitals in Mufindi: in Mufindi, 1 hospital was private). Moreover, five of the HFs identified for study were HCs, the remaining were dispensaries. Two out of 3 HCs available in Mkuranga district were included in the study, and both were public, their selection being non-random. One HC, namely St. Vincent HC belonging to the RC Church was not included purposely for reasons explained above or those explained before [18], including that of having been less utilised by community members to allow a sufficient sample for the study within the planned study period. In Mufindi, 3 out of a total of 5 HCs were also included based on random selection. The dispensaries selected for study in both districts were identified randomly, totaling 5 in Mkuranga and 7 in Mufindi [18].

As for the place of work, 50 out of all 78 HWs who participated in the study were found working at public HFs, 13 working at HFs operated by the FBOs and the remaining ones were working at commercial HFs. Selection of study participants depended on the availability and willingness of the approached HWs to participate. Sample size calculations and randomization of participants were found not being necessary all the time because comparative statistics was not in focus in the present study. Random selection of 2-4 participants was only performed where more than two, and at least four willing HWs were identified at an involved HF [29].

### Data collection methods

Data from the HWs were obtained through interviews using a standard questionnaire administered by trained research staff. The questionnaire comprised a mixture of closed and open-ended questions carefully designed. Open-ended questions were aimed at gathering qualitative data to augment or supplement the answers to closed questions. The latter type of questions was aimed at gathering the data for which a quantitative analysis would be performed to indicate mainly the frequency distributions of the responses/answers under each of the selected questions. For some of the questions, multiple responses were allowed and these were later coded for quantitative analysis. The first round of the interviews was conducted between November 2005 and October 2006 [29]. The topics covered related to respondents’: knowledge and perceptions about fANC guidelines and service package; adherence or non-adherence to the guidelines, including administration of SP and the underlying motivations. Other issues included reasons behind observed levels and coverage of IPTp-SP utilisation as well as relationships between IPTp-SP services, fANC guidelines and general government policy on ANC services. To support or challenge the views obtained from the frontline HWs, the investigation made reference to additional views obtained through the informal interviews made with the ANC clients using the same HFs as elaborated elsewhere [18,36]. This mainly relates to issues of availability of essential drugs, ANC cards, HW’s efficiency in delivering quality services, staff-client interactions and perceptions in relation to IPTp-SP benefits, accessibility and knowledge.

### Data processing and analysis

Qualitative data were analysed manually by adopting a qualitative content analysis technique. As described elsewhere in a sister paper on data from the ANC users [18], as well as ANC givers [29], this involved a process of organising the information bits (meaning units) in themes according to study objectives and, subsequently for easy comparison and triangulation. The process also involved reading through the contents of the stuff and jotting down the key points without aid of computer based software [18,36]. However, some of the qualitative data could be coded to allow quantification in the analysis for tabulating simple statistics where possible for doing some comparisons [29]. Either a Pearson’s Chi-square test or Fisher’s Exact test techniques was applied when performing cross tabulations in attempt to find out if any of the observed difference was statistically significant. The Fisher’s Exact test was applied for smaller samples of less than 30 units while Chi-square test was used for relatively larger data samples [39]. The probability parameters (P-values) from the statistical tests performed are presented only where they appeared to make any meaningful message in line with the study objectives and scope of the present paper. Most of the detailed analyses so performed did not indicate meaningful results for presentation in this paper mainly due to small sample nature of the data collected, especially considering number of the HCs and hospitals and a few dispensaries. That is to say that performing an analysis which could lead to meaningful comparison of the HWs’ opinions by designation and other socio-economic characteristics of the respondents’ cadres, number and places of their work within each district and between the two districts, as well as their duration (number of years) at their work stations or in the health sector could not make sense. At several dispensaries, it was observed that a few HWs found showed a skewed HW distribution when considering the different cadres of staff: some of the facilities were mainly or entirely manned by female or male staff either all days or at least for some of the study days. As the answers for some of the questions were missing, the frequency shown in the results section were calculated based on only the responses obtained under such questions (and are abbreviated as ‘n (%)’ rather than N (%) for the total sample of the interviewees). Care was taken when it came to interpretation of responses to open-ended questions because of observed inconsistencies (during the pre-testing of the tools phase) in the way respondents answered related questions. Interviewers were therefore trained to make reference to previous and potentially contradicting answers during each interview as it deemed necessary while administering the questionnaire e.g. by going back and forward including repeating some of the questions for confirmatory purpose. Thus, it may seem perplexing to see some of the answers/results overlapping and the open-ended nature of the study questions contributed to this happening apart from the recall bias of the respondents.

### Ethical considerations

The study conformed to all aspects of the Helsinki Declaration. The participants approached were informed of the study objectives and major ethical considerations including the participation on voluntary and non-payment compensation terms, anonymity of the participants, confidentiality of some of the information they gave, the expected benefits from the study including sharing the study findings with the policy/decision makers at district level and national level (including the NMCP officers) and international audiences interested in reading about malaria control issues/challenges in Tanzania for possible corrective actions or recommendations. Participants were also assured of no penalty or other negative consequences if they decided not to participate or if they withdrew from the study anytime as they would have wished to. Those who accepted to participate did provide their consent in writing. The national ethical clearance for the study was granted by the Medical Research Coordinating Committee [29,36].

## Results

### Duration at workplace

Each respondent had been working at the HF where he or she was recruited for the study for at least six months. The majority had been working there for more than 2 years, and some of them e.g. nurses, midwives and medical attendants (and not many of the clinical officers) had worked either at the study HFs or elsewhere within an ANC system for more than ten years. Hence, these had already gained sufficient experience with the health seeking behaviour of the ANC clients. The mean number of years of working at the HFs of recruitment was 8.3 (range: 0.5–24 years) and this was considered adequate for the respondents to recall their experiences on issues under investigation.

The composition of interviewed HWs included clinical officers (COs), nursing officers (NOs) and other cadres of nurses such as public health nurses (PHNs) and midwives; maternal and child health aides (MCHA), health assistants and medical attendants, previously known as nurse auxiliaries [29]. Out of the 35 HWs interviewed in Mkuranga, 20 (57.1%) were working at public HFs, the rest at private HFs. In Mufindi, 30 (69.8%) out of 43 HWs were working at public HFs, the rest at private HFs. Overall, 51 (65.4%) of all respondents were working at dispensary levels and 62 (79.5%) were females. COs and senior NOs are normally not involved in the provision of clinical services at the ANC clinics, but occasionally assist the clinical staff due to general staff shortages. Otherwise, the midwives, PHNs, MCHA and medical attendants are mostly involved in the provision of ANC services [29].

### Knowledge about ANC guidelines and services

#### Antenatal care guidelines

Respondents’ knowledge about ANC guidelines related to their awareness of general issues as well as specific components, elements and characteristics. Thus, the notion that government policy recommends basic ANC services to be delivered free of charge was expressed by 17 (49%) of all respondents, the perception being that all services should be given for free irrespective of one’s income and other social statuses. Awareness of the existence of fANC guidelines was expressed by about three quarters of the respondents in Mkuranga and by four-fifth of respondents in Mufindi. These HWs reported to have heard about the fANC guidelines from participating in courses or from receiving information from superiors during health service supervision or other kinds of in-service orientation. Although some respondents stated to have read fANC documents, including those with guidelines on ANC delivery, still many could not explain the content well. But, after being probed to identify specific elements of the fANC guidelines, the majority could identify IPTp-SP as one of the central components/elements. Less than 50% of all respondents could state that “providing ANC clients with health education on MiP control” was a fANC service. Meanwhile checking foetal movement and growth, undertaking palpation, administration of medicines such as ferrous/folic acid, mebendazole and vitamin-supplementation tablets, as well as blood checking/test for haemoglobin (Hb) and measuring blood pressure were reported by less than 10% of the respondents from both districts, each. It was discovered through informal communication with the staff concerned after the interview sessions that failure to mention these elements by majority of respondents did not mean that actually the respondents did not know such elements and instead they either forgot to mention them or did not see the direct relationship of such elements with the fANC guidelines/requirements.

As shown (Table 1), the number shown outside the brackets for each of indicator represents the number of HWs who stated the respective indicator as their answer to the question requiring them express their knowledge about the key elements of fANC guidelines. The proportions/percentages of the HWs who mentioned the respective element/indicator out of the total number of the HWs interviewed per district numbers are as shown inside the brackets. It can be observed that only about half of the respondents considered the IPTp-SP strategy to be a preventive intervention as opposed to a curative intervention for treatment of confirmed malaria. ITNs were mentioned by about half of the respondents as an essential preventive intervention against malaria infection, as stated in the fANC guidelines.

**Table 1:** Knowledge about selected elements of focused ANC (fANC) guidelines among interviewed HWs who confirmed and verified their knowledge about fANC in Mkuranga district and Mufindi district

**Element of fANC guideline**	**Mkuranga district (N = 35)**	**Mufindi district (N = 43)**
1. Being aware of (familiarity with) the fANC guidelines	27 (77.1)	36 (83.7)
2. Knowledge that government policy recommends basic ANC service delivery free of user fees	17 (48.6)	21 (48.8)
3. Knowledge of IPTp-SP as one of the elements of fANC	31 (88.6)	38 (88.4)
4. Knowledge that giving health talks/education on MIP issues to clients was one of the required fANC procedure	8 (22.9)	14 (32.6)
5. Knowledge that SP given for IPTp is intended as a preventive treatment unlike it is used for treating patients with confirmed malaria	18 (51.4)	25 (58.1)
6. Knowledge that ITNs use is recommended in the fANC guideline/guidance	21 (51.3)	21 (48.3)
7. Knowledge that at least two doses of IPTp-SP is recommended in the fANC guidelines	27 (77.1)	38 (88.4)

#### Recommended doses of IPTp-SP

Knowledge that three tablets of SP were recommended per standard dose for IPTp was expressed by 30 (85.7%) of the respondents in Mkuranga and by 43 (100%) of the respondents in Mufindi. Moreover, knowledge that at least two doses of IPTp-SP were recommended during scheduled ANC visits was verified by three quarters and four-fifth of the respondents in Mkuranga and Mufindi, respectively. In Mkuranga, 5 (14.3%) of the respondents reported that the administration of three doses of SP for IPTp was appropriate. Such respondents revealed to have received instructions from trainers at seminars organized by a non-governmental organization as well as seminars attended either by respondents themselves or by officemates at HF level. Equivalent information was obtained from superiors during routine ANC service activities at HF level. Such dissemination of recommendations for IPTp-3 administration was confirmed by members of the district Council Health Management Team (CHMT) dealing with RCH issues. Yet, both HWs and CHMT members questioned whether this practice (of providing IPT-3) was in line with national IPTp guidelines, as they generally doubted. Participants were of the feeling that the national guidelines still emphasizing on the administration of two doses of IPTp with SP while other organizations recommended more than three doses whenever conditions allow needed further inquiries to confirm which was which in order to avoid the blames that might fall upon the frontline HWs who strictly adhered to the existing instructions either those given by government or by a non-governmental authority. This happened before the WHO has given its stance to clear the prevailing doubt or confusion about the number of doses to administer. Now it is clear that more than two IPTp doses can be administered [5].

Additional views were expressed regarding confusion seeming to prevail regarding the understandability of the national fANC guidelines. For instance, HWs reported being confused with what to do in terms of providing IPTp-1 to the pregnant women registering at ANC clinics either before the officially recommended 20-24 weeks of gestational of pregnancy. Confusion was also reported about the clients who came after the officially recommended 28-32 weeks gestational age for administering IPTp-2. Another confusion related to the recommended one week spacing/between administering ferrous/folic acid and SP and how this relates to gestational age of the pregnant woman. Some HWs confessed to have been administering SP and ferrous/folic acid tablets concurrently and claimed that this was done purposefully to the clients who were found to be highly anaemic and therefore seeming urgently needing iron. The confessing HWs seemed to be uncertain of whether or not they were right when providing the two treatments concurrently and suspected that doing so might be against the official guideline specifications. Another dilemma such workers reported to have been faced with is that of their common practice of allowing at least some of the client to take SP unsupervised at home, and thus risking those who did not comply. Finally, confusion was expressed about what to do in terms of administering SP to a pregnant woman confirmed or suspected to be suffering from a malaria attack. This was particularly complex when the malaria attack occurred at a gestational age earlier than the recommended period of receiving either the first dose (e.g. before the week 20^th^ week of pregnancy) or later after the recommended period of administering the second dose (i.e. after the 32^nd^ week) for safety reasons. As reported, this was a great challenge not only to the frontline HWs, but also the clients who were already aware of the risk of taking SP at very early or very late stages of pregnancy. During the interviews and informal discussions with a number of clients at HF and community levels, these concerns were also expressed. The HWs in the present study case also felt the urgent need for the health authorities to recommend a drug alternative to, or that would replace, SP for IPTp, if possible and as immediately as it allowed. They based their claim on the argument that this was important in favour of the clients who report or are clinically confirmed to be allergic to SP and for whom the use of ITNs was so far still the only option officially recommended.

#### Timing of the first and subsequent doses for IPTp-SP

Over 85% of HWs interviewed knew the recommended gestational age for administering the first and second doses of IPTp-SP. Administering IPTp-1 during the 20-24^th^ week period was recognised by 33 (94.3%) of the respondents in Mkuranga and by 39 (90.7%) of the respondents in Mufindi. Moreover, 25 (71.4%) and 38 (88.4%) of the respondents in the two respective districts knew that the 2^nd^ dose of IPTp-SP was recommended during weeks 28-32 in the national fANC guidelines. The remaining respondents did not know this. Statistically, no significant difference were observed in respondents knowledge about the gestational age for administering IPTp-SP between the respondents of different sex, age, HF affiliation, designation/cadre and years of working experience. Among those who knew the gestational ages appropriate for IPTp administration, 50 (64.1%) were working at public clinics whereas 28 (35.9%) worked at private clinics.

Several study participants asked for clarification from the present study investigators on whether or not the SP given for IPTp was meant for prevention of malaria and, if so, whether it really worked as an efficacious/effective and safe drug. They could not understand the reason for taking SP tablets without prior malaria diagnosis and wondered why SP was used for both preventive and curative purposes since to them using a medicine of any kind such as anti-malaria tablets means to treat the infections.

### Psychosocial and cultural barriers for IPTp uptake

#### Beliefs and traditions affecting pregnant women’s registration for ANC services

Young lactating multigravidæ were the group leading to recognise their pregnancy status at a late stage. This was reported by 29 (37.2%) and 20 (25.6%) HWs in Mkuranga and Mufindi, respectively. Systemic factors perceived by HWs to contribute to late registration of pregnant women to ANC clinics are presented (Table 2). In this Table case also, and as highlighted above under the methodology section, the number shown outside of the brackets stands for the number of HWs who gave the respective answer to the question requiring them to identify the reasons/factors influencing pregnant women of different gravid experiences to attend clinic late. On the other hand, the numbers shown in brackets indicate the proportion of the HWs who mentioned the respective category of the factor/reason to the total number of the interviewees.

**Table 2:** Reasons for late registration for ANC by pregnant women as perceived by frontline ANC staff in Mufindi and Mkuranga Districts based on data for both districts combined out of all interviewees (N = 78)

***Perceived reasons for late booking***	***Primigravid*** **æ**	***Multigravid*** **æ**
	**n (%)**	**n (%)**
i. Fear of being seen of pregnancy by envious or mocking community members	35 (44.9	7 (9.9)
ii. Long travel distance to ANC clinic	26 (33.3)	35 (51.5)
iii. Carelessness and negligence of pregnant women or their family decision-makers	2 (2.6)	52 (66.7)
iv. Ignorance of health risks of late booking	18 (23.1)	4 (5.1)
v. Perceived or real cost barriers (e.g. user fees, ITN voucher redemption, and transport)	16 (20.5)	8 (10.3)
vi. Family/domestic occupational and other social commitments	14 (17.9)	21 (26.4)

A registration is officially considered to be late when the first ANC visit is paid during week 20 or later in pregnancy [40]. Late registration was viewed as being common among primigravidæ as indicated by 30 (85.7%) and 37 (86.0%) of the respondents in Mkuranga and Mufindi, respectively. Late registration was also considered a common behavior among the multigravidæ as reported by 33 (94.3%) and 35 (81.4%) of the respondents in the two districts, respectively. Pregnant women’s irregular and untimely attendance to clinics was reported to limit access to ANC services. It was lamented that healthcare seeking behaviour in both districts is culturally rooted meaning that cultural values have an important influence on people’s health care seeking behavior and sometimes their perceptions about health and health related problems. Visiting ANC clinics in early pregnancy is commonly considered unrealistic or undesirable because these women would be spending substantial time (on transport and waiting for health services). Spending time longer than necessary at HF level was claimed to disappoint some women since the time lost would otherwise be used for domestic purposes. Furthermore, pregnant women could be looked upon by the society around as being boastful when revealing their pregnancy, hence eliciting envy, jealousy, social exclusion and even bewitching would be experienced.

For a large proportion of pregnant women the first point of contact to pregnancy-related services is with a local traditional birth attendant, the practice/behavour which contributed to delay women contact to ANC clinics as reported by 6 (7.7%) and 2 (2.6%) respondents in Mkuranga and Mufindi, respectively. Multigravidæ, in particular, were reported to ignore the benefits of ANC services and the risk of pregnancy-related complications as compared to the primigravidæ. These women tend to rely on support provided by TBAs and may become aware of, and react to, otherwise preventable complications too late. Similarly, reports were given about some pregnant women delaying to attend clinic if they lacked money for redeeming the discounted vouchers for ITNs that are usually offered to the ANC clients. At times the ANC clients concerned were directed by the formal HWs to follow the price subsidised ITNs at the accredited retails sources or particular clinics. This limited the women from poor families who had no cash money to recover/redeem or had no money to be spent on paying for transport when needing to travel long distances. This was similarly reported during interviews and group discussions with the current and recent ANC users [18].

#### Occupational commitments affecting ANC utilisation

Pregnant women’s family and household commitments were reported to pressurise women and negatively influence their ANC utilisation by delaying registration and/or disrupting scheduled visits. Domestic activities include cooking, collection of firewood, fetching water, taking care of children and attending family-related social events (e.g. forty-night mourning/grieving periods in the event of deaths among close relatives). Other activities include farming obligations related to land preparation, planting, weeding and harvesting of crops. In Mufindi, in particular, women spend substantial time on farming activities in efforts to raise their family and personal cash incomes. In Mkuranga, women commonly spend days in the forest preparing charcoal for retail businesses and forget or ignore to attend clinic as scheduled. In both districts respondents reported that during particular times of the year (e.g. when planting maize and rice, weeding, chasing away rice-eating birds and harvesting) pregnant women were clearly more reluctant to comply with their scheduled ANC visits than during less busy times of the year.

The HWs in both districts also shared additional experiences regarding other factors seeming to influence primigravidӕ develop feeling of shyness or fearing to expose their pregnancies immediately. Consequently, such women delayed or failed to contact the ANC clinics. The factors for this happening mentioned include: some pregnant women conceiving in their late ages (e.g. mid or late 40s); those conceiving while they are still lactating and within a short period after giving birth; teenagers fearing to be expelled from school or families if they found to be pregnant; women who come to discover that they have carried the pregnancies belonging to men other than their known spouses/husbands. Some of the factors seemed to influence the primigravidӕ more than the multigravidӕ and vice versa (Table 2).

HWs reported that it was not uncommon for pregnant women to deliberately stay away from or postpone ANC services due to fear of side-effects of SP on the foetus. Meanwhile, such respondents were faced with the dilemma between moral obligations of informing clients about possible side-effects of SP (most commonly observed during the first trimester of pregnancy unlike in the last stages as commonly known before [5] and the negative implications of such information on the acceptability of IPTp-SP services among pregnant women.

Verbal reports from the respondents revealed that the clients with a higher level of education (e.g. teachers) were more reluctant to accept SP administration in fear of side-effects. Such women claimed to have been hearing the announcements given to the public through, for example, the media, which according to the majority of the participants were greatly amplified. Health education was reported by the frontline HWs as most often being provided in form of group sessions involving pregnant women at the clinic level in the morning hours. Therefore, those who hate waiting for a long time in queues at the congested ANC clinics in morning hours were deciding to arrive at the clinic very late e.g. around noon or late afternoon, hence missed the opportunity for either receiving health education completely or if they got it partially. Tendency of the women to arrive very late at clinics when the HWs were already tired tempted the staff concerned to rebuke the clients concerned. Some of the HWs responding to this study expressed their dissatisfaction with a few of the staff who behaved so since they distorted the image of other caring (or honestly ethical) staff and the image of the national/district healthcare system at large. As a result of such few HWs’ poor client handling behaviour, a number of older pregnant women felt being too old to be rebuked like children especially by the younger HWs, and this made such clients feel being humiliated, and unnecessary to comply with revisiting the ANC clinics as scheduled and indicated on their ANC cards.

In Mufindi, practising DOT for IPTp-SP was challenged by a high frequency of pregnant women who arrived more or less drunk at ANC clinics. Drinking local alcoholic brews made of sorghum or millet (locally known as *komoni*) and juices of bamboo plants (locally known as ulanzi) is culturally accepted even among youth and pregnant women. HWs speculated that some pregnant women might deliberately drink before visiting ANC clinics knowing that they would be excluded from SP administration. Instead, HWs would have to consider leaving SP tablets with the clients to be swallowed at home. This was acknowledged to compromise compliance and to negatively influence the clients’ health behaviour in general. Practising DOT was also reported to be hampered by other factors such as shortages of clean water and cups at HF level and that having disposable cups would help to motivate the clients who hate sharing cups between successive users at HF level. That is, many ANC clients were said to dislike the sharing of cups with other clients for taking SP tablets under DOT at the clinics levels. This was considered being even worse when regular cups were not available and instead empty drug containers were used as substitutes.

#### Early registration at ANC clinics and uptake of IPTp-SP doses

Early registration at ANC clinics was reported to be a common behaviour among the pregnant women who: (i) appreciated the benefits of a full course of ANC services; (ii) sought early consultations to confirm their suspected pregnancy with the intention of performing an abortion or stopping breastfeeding; (iii) wanted to undergo early testing for HIV infection to determine if they would qualify for the prevention of mother to child transmission services; (iv) wanted to receive an ITN voucher as early as possible to allow them to protect their pregnancy against mosquito bites causing malaria; (v) feared reprimands by ANC staff if postponing (or delaying) registration; (vi) feared the culturally embedded shame of infertility and thus anxiously needed to know her pregnancy status; (vii) were pressurised by their husbands/spouses to register early for ANC services despite personal unwillingness. Finally, HWs occasionally discovered pregnancies among clients receiving other services (e.g. while consulting an outpatient department or attending a school health programmes) and using this opportunity to convince the clients concerned to register to ANC services immediately.

### Other health care system constraints

#### User fees for ANC services

Despite the official policy of free ANC services in public HFs in Tanzania, some HWs reported that user fees for ANC service were claimed to exist and that such fees excluded or delayed the poorest women from seeking ANC services. In Mkuranga and Mufindi, 11 (31.4%) and 7 (16.3%) of the HWs, respectively, confirmed the existence of user-fees for on selected ANC services such as pregnancy testing, ANC registration cards, laboratory screening and medicines for certain health conditions.

Stock-outs of ANC cards were considered being a common occasional problem, mainly at private HFs or HFs operated by FBOs. In effect, photocopies of ANC cards or notebooks had to be procured by the clients at a price of up to 100-200 shillings (USD 0.1-0.2).

Some HWs were of the opinion that free ANC services was recommended for public but not private HFs. This opinion originated from the observations that the district and national health authorities paid little attention to satisfying the needs of the private health sector providers in terms of supporting them with the supply of essential drugs like SP and other materials useful for satisfactory delivery of ANC services when the HFs concerned cannot meet all needs. It was claimed to be an embarrassing situation to frontline HWs working in the private sector when experiencing angry clients complaining about the costs of services thought to be free. Failure of clients to pay user fees at ANC clinics operated by FBOs or the private sector was reported by 9 (25.7%) of the respondents in Mkuranga and by one respondent in Mufindi. Critique of the national user-fee exemption policy was expressed by 22 (62.9%) of the respondents in Mkuranga and by 24 (55.8%) of the respondents in Mufindi. The most common argument was that service delivery is costly for the society and medical and material supplies to the HFs are often inadequate.

#### Stock outs of SP and other supplies related to IPTp uptake/delivery

Further on the issue of supply stock-outs, data showed that HWs at private HFs, in particular, experienced stock-outs of SP and other essential supplies required for free RCH services. It was commonly viewed by the respondents from both sides – private and public sectors that central and district health authorities did not give the private sector providers the same priority as public sector providers on important health services support challenges. This includes issues relating to staff training, essential supplies and supportive supervision of the frontline HWs especially those working in remote rural or peripheral settings. In both districts, it was lamented that private sector providers were suspected for charging the clients for drugs and other supplies received for free service delivery by public health authorities. In the case of stock-outs of SP at HF-level, clients were often advised to procure SP in the private market despite uncertain availability and poor quality control related to accreditation of manufactures expiry dates, qualifications of the vendors etc.

## Discussion

### Knowledge issues

In the case of knowledge in relation to IPTp guidelines, the present study reveals the IPTp strategy in both districts reflected a wider chance for being implemented effectively when the HWs were adequately informed on how to appropriately use the national guidelines. Knowledge about fANC in general and IPTp in particular was inadequate, in spite of the seemingly to be moderate-to-high awareness on some elements of it among the respondents. The confusion about the ambiguities of the national fANC including IPTp guidelines e.g. risks of administering SP for IPTp, timing of the first and last doses, timing and dosage required for the administration of folic acid tables to women eligible to use SP for IPTp, and other directives, indicates that HWs sensitisation or training/orientation on the issues has not been so far adequate in the national health care system. The view that administering IPTp-3 would confuse the frontline HWs if it was emphasised as instructed was also shared by the national level officers [17]. In contrast, some officers at district level challenged this view by claiming that both the WHO and national fANC guidelines impliedly allowed more than two doses when they use the words ‘at least two doses should be administered’ , and that it was made clear that if the woman concerned had began visiting ANC clinic early and then continued attending clinic as scheduled [41]. It was until 2012 the WHO came to make it clear that the administration of IPTp-SP even nearest to giving birth was still safe and allowed, together with other clarification on the use of folic acid to pregnant women needing IPTp at the same time [5].

While as said before most of the issues relating to IPTp administration have been worked out already at global level [5], the most key message here is that it should not be taken for granted that as long as the guidelines are instituted, every frontline HW can easily comply with such guidelines by finding them to be understandable. The observed variations in the knowledge about fANC and IPTp issues among the present study respondents was possibly contributed by the different opportunities the respondents concerned had to participate in seminars/workshops or and the different opportunities obtained by the staff in terms of being supervised by the senior officers at HF or from the district levels [29]. This reflects the need or continued searching and implementing programmes for informing the service providers of all updates relating to their job and this includes training programmes for HWs. Confusion about how to implement the IPTp guidelines among other fANC services and later on improved HWs performance following training on effective use of such guidelines was noted in the neighbouring country of Kenya [42] and various authors have recommended training to orient frontline HWs as an important contribution to improved operational effectiveness of novel interventions such as IPTp [3,16]. Reviewed, the original national fANC guidelines, however, seemed to clearly suggest the administration of SP for IPTp doses anytime after the 16^th^ week of pregnancy as long as there is an interval of one month apart [14]. Even the WHO has come up with a recommendation that SP can be used or provided to pregnant women for IPTp in moderate to high transmission settings at each of the scheduled ANC visits, thus as said several times, this means that the administration of more than two doses was acceptable [5,23]. Thus, the frontline HWs concerned might not have been able to understand what the words ‘at least two doses’ really meant literally and even spared enough time to consult for clarification, leave alone the fact that even their superiors/supervisors were unequally informed of what the words meant. Otherwise such superior officers would possibly have taken measures to strictly ensure that the frontline HWs were complying with the guideline, and this was a feeling of the national level officers also [17]. The issue raised about whether using IPTp-SP and ITNs together effectively was still beneficial or would offer any added benefit gives another key message to the NMCP authorities responsible planning health communications to the general public and service providers implementing the national guidelines. Indeed this is one of the practical challenges scientists around the world which have been confronted when discussing the possible most optimum ways of scaling up use of IPTp in combination with ITNs. Meanwhile, scientific and policy community also are still faced by a critical question on whether there is any drug alternative to, and to replace, SP for IPTp [2,25]. So far, studies are still underway looking at some alternative drugs e.g. mefloquine [5].

The prevailing confusion or dilemma about SP administration for IPTp also reflects how some of the staff who succeeded to attend the fANC related seminars could even not comprehend well the teachings of their trainers if at all the trainers themselves were capable of taking their teachings as seriously as they necessary. Furthermore, it is evident that some of the present study respondents could not give correct answers on why the IPTp strategy was recommended. Those who asked to be given assurance on whether SP really could prevent malaria rather than being used as a curative drug against the confirmed parasitemia reflect the limited knowledge existing in the community of health care service providers.

### Psychosocial and cultural factors influencing ANC attendance and IPTp uptake

#### Beliefs about and attitudes towards SP

From the present study, it can be seen that the late registration shown by some of the pregnant women at the clinics has been contributed by a combination of factors. Some of the factors are within clients’ themselves (psychological) while others are external in that they originate from the environment around the pregnant clients in their respective community and HF settings. The previous report indicate that even professionals doubt about the safety of generic antimalarial drugs including SP [4,25,43,44], and such reports are consistent with the views expressed by some present study respondents. It can also be observed from the present study that some pregnant women seemed to be negligent by not adhering to the recommended ANC visits or taking SP for IPTp. Possibly, it is not necessary that such women were poorly informed of the benefit of complying because as human beings, such women might have had their own feelings/perceptions about the suggested visits and drug uptake even if they would have been properly and adequately informed of not complying or the benefit of complying. At times, people prefer to exercise personal freedom by behaving the way they like regardless of any information they may have about the risks associated with their behaviour. Therefore, changing one’s behaviour is not easy, or abrupt, or over night, process. The reported negligence by some of the pregnant women to visit ANC as scheduled as noted in the present study are consistent with reports on similar situation happening elsewhere in SSA, the key factors for this tendency include: the social stigma about unwanted pregnancies or fear of reprimands from service givers for various reasons, ambivalent towards others if they notice one’s pregnancy, wary of seeming boastful if attending clinic early or spreading the pregnancy news openly, and actual or perceived cost of accessing the service, and staff behaviour when they interact with their clients at HF level [16,27,35,45,46]. Thus, pregnant women may feel to be more comfortable if they allowed to act independently including when making decision on whether, where and when to seek pregnancy care. Therefore, subjecting such women to swallow the medicine under DOT without their will may seem forceful and disrespect of their freedom to opt for using or not using health care, and to some of them this is intolerable/unacceptable. This means, the HWs dealing with such clients need to be competent enough at advising and persuading their clients through health education and counseling schedules. The activity of health counseling is time consuming especially if one meets a client who is either too critical to the feedback given or difficult to capture/comprehend the messages. It may cost the HW meeting clients of this type if the HWs concerned do still have to spend more time with other clients while other duties that are pending, leave alone such staff sometimes entering into crash with their clients.

On the other hand, the reported fear about possible side effects of SP affecting uptake of the IPTp doses, protective efficacy of SP and its added advantage if one is effectively using ITNs are a challenge to NMCPs officers and frontline workers. This finding reveals that community education about SP-IPTp is still required and should be a continuous task. Pregnancy is a sensitive issue which tempts the women concerned hesitate or avoid consuming particular things and this includes medicines because they may fear from facing the abnormalities leading to abortion or other disabilities to the foetus or newborn baby or the pregnant mother.

The results presented show that the more educated ANC clients seemed to be more critical and reluctant to take IPTp-SP and this indicates an important challenge to health education designers. The latter finding may sound strange since evidence continue indicating mainly a positive relationship between the clients’ level of education and their ability to comprehend the health messages and more chances for them to seek appropriate RCH services [35]. Possibly, those seeming to be reluctant to take SP in the present study case were influenced by the rumours about possible negative treatment outcomes if the SP were improperly administered. Scientists warn that SP should not be administered concurrently with cotrimoxazole given their redundant mechanisms of action and synergistic worsening of adverse drug reactions [5,47]. However, neither the present study nor any other studies have attempted or succeeded to evaluate whether the HWs comply with this guideline and in case not what inconvenience this might have caused in the community. Social science experts guided by health belief and self efficacy models/theories hold that sometimes the more a person is learned the more he or she becomes sensitive of knowledge related issues and therefore a person becomes more inquisitive or suspicious if not well informed and convinced [29]. Thus, adult and sound minded people can only be persuaded to accept taking medicines only if they trust in its treatment outcomes especially its safety. It is very important that even the service providers were found to be less properly informed on the usefulness of SP including its safety during pregnancy, so they might be part of the community opposing SP’s use if they share the same sentiments as their clients.

The issue of DOT compliance has been looked at from the service providers’ perspective while ignoring the role played by the service clients. We argue that it is difficult for the HWs as shown from the present study results to reject their clients’ request to take SP at home on grounds of having empty stomachs or when they claim to be physically exhausted after walking long distances from home and those presenting claims of being allergic to sulphur compounds. In the absence of knowledge on the safety of SP even if used by a hungry/starving client [5] or in the presence of claimants of facing allergy with SP, it may not be realistic to blame the HWs who agree with such clients.

#### Shortage of SP, HWs and water cups at HF level

The reported issue of cups shortage making some women feel unhappy to share a few cups while taking SP under DOT for IPTp at the clinic level, could be addressed by asking the clients concerned to bring their own cups/vessels from home for that purpose if the health system cannot be able to budget for procuring the disposable cups. In contrast, the problem of shortage of SP occasionally at HF level is a systemic challenge which should not be left to be chronic or continue since this is likely to constrain further the coverage targets. Consideration should be made for the women living far away from public HFs and therefore being forced to consult private clinics. It is very unfortunate as revealed by the present study findings that private sector providers do not always receive government support/subventions in terms of SP and other supplies for free delivery to the clients, apart from the vaccines against several immunizable diseases. So, how can universal accessibility or coverage be achieved or ensured without stern measures being taken to address this problem? There are rural places in Tanzania whereby private sector providers operate more than public sector providers in Tanzania [48], therefore, inequalities may prevail between regions and even between areas within the same region/district whereby people access public and private HFs differently. On the other hand, failure to supply SP to both government and private HFs may result from failure of the government authorities concerned to present appropriate plan and budget request for grants from the Global Fund for Fighting AIDS, Tuberculosis and Malaria (GFATM). Although the plans in place seem to indicate a number of issues to be addressed e.g. training frontline HWs and making important health procurements using chance given to Tanzania to receive grants from other sources such as The US Presidential Malaria Initiative [15], the issue of SP required for wider distribution to include even the private HFs might have been overlooked. This might be due to the government authorities concerned paying relatively a greater attention to the more expensive medicines e.g. newly recommended artemisinin combination therapy (ACT) used as first line drug for malaria treatment and the antiretroviral (ARV) drugs used for PMTCT services than it does on SP for IPTp. Nevertheless, some of the district and national level authorities opposed several of the allegations related to private sector providers not being supplied with essential health care related materials although they admitted that occasionally stock-outs of SP were experienced. The views of such officers was that the stock-outs episodes were not a so serious problem and it was contributed partly by the failure of the private HF owners/officers to notify the authorities concerned, as documented in more details elsewhere [17,29,37].

### Strengths and limitations of the study

In spite of several variables shown in the present paper being represented by small sample sizes, there are meaningful inferences to be extracted as guidance to future researchers who may need to design questionnaires covering the aspects/elements indicated and for comparative purposes between areas and among the staff with different characteristics. Generally, this study provides additional evidence supporting previous reports from other studies in Tanzania and abroad, some of which have been cited under the background section of this paper. There are many good lessons the NMCPs can learn from this study, as they do from such other programs as nutrition and expanded programme for immunization (EPI). Also, the study timing period was appropriate since it provided a good room for coming up with the findings that could guide malaria experts at national and global level to identify workable recommendations based on current/observed HWs’ knowledge, perceptions and practices in relation to existing guidelines and facilities. Evidence shows that local discourses and HWs’ comments do influence concerns about MiP interventions [21,35]. It is interesting that some of the views presented by the HWs in the present study and as documented elsewhere based on the same study respondents [28] are mainly supported by the opinions of the current and recent ANC service users, particularly on issues related to direct and indirect costs and a few elements affecting the quality of care [18]. More findings will appear in the next papers currently under preparations and this helps to avoid keeping the present paper longer than it is now.

The present study is subject to several criticisms as follows: where the data for each district were stated separately, it would have been more convincing to present sample sizes of the variables and staff presented which are large enough to allow more detailed statistical analysis including multivariate and principal component analyses. As the data are at present, one only guess about what the results might be if certain variables e.g. staff cadres and HFs had larger sample sizes that could be analysed comparatively to explain the situation much better. This remains an open question for guiding future researchers interested in undertaking studies on same/similar aspects. Other limitations are related to representativeness and biasness of the study, and these mainly include: i) the papers’ focus on the experience and views of the HF based staff and from two districts only, hence showing a few experience-based challenges/views from the service users; (ii) use of convenience sampling strategy to select several HFs and HWs; (ii) some respondents failing to understand well some of the question posed and critically thinking to give the answers they might have believed to please/influence the researchers take the message to the government authorities concerned! This is possible since a larger number/proportion of the respondents were those of low ranking staff cadres. Many of the latter cadres had never attended special courses on fANC and MiP aspects [28]. There was unequal and yet small sample size representations and the nature of the data obtained [29].

### Conclusion

This study reveals that in general the frontline HWs in the study districts were positive to the malaria IPTp policy. However, they were mainly worried of psychosocial and some systemic (mainly supply related) factors lowering the chances for the eligible pregnant women to attend clinic and eventually take IPTp with SP doses. Although removing such worries could not be solved overnight using a single approach, there is an obvious need for strengthening a multi- and intra- sectoral and disciplinary problem-tackling approach to address issues relating to teenage pregnancies, late ANC registration by the women of low and high parities, shortage of essential supplies such as drinking water and water cups (e.g. use of disposable cups that are cheaper), importance of child spacing, as well as enhancing people’s knowledge about the risks of MiP and IPTp in particular. Tanzanian media are commended for publishing or announcing news about safe motherhood programmes initiated and implemented by the MoHSW in partnership with other governmental and non-governmental agencies, and among the efforts shown, sensitization of the community on pregnancy care and male involvement seem to continue taking an important position in TV and radio programmes. More concerted measures to ensure that pregnant women can access basic health services close to their homes should continue being taken, as this will help to motivate the potential users of the desired and available services. Systemically also, the HWs should receive regular supportive supervision, supplies of and orientation on fANC guidelines. The guidelines should not be just supplied to them at HF level and left on desks or in shelves, but also be effectively used for reference when delivering the service. Such guidelines should also clear, non-misleading/confusing or unambiguous and this means several statements appearing in the guideline to highlight on same issue should not be inconsistent and the steps should be easy and practical to follow; HWs training on malaria control issues should not a one-off process, and the trainers should be aware that even after being oriented at seminar levels or during the routine health service supervision schedules, the HWs tend to forget some details or procedure. Thus, refresher training programmes help to update/remind them, and this is important especially when new interventions are introduced. Even when the old interventions prevail, a continued research on the practical aspects of whatever intervention in terms of logistics and its acceptability to target service providers and users should be an essential and actually integral element of needs assessment and monitoring other issues around its current existence and sustainability. Meanwhile, health education messages associated with campaigns for ITN use for malaria prevention among the pregnant women and under-five children should contain all the key messages aimed at enhancing the degree of understanding of the target audience regarding the mutual benefits of using both ITNs and IPTp in an integrated manner. Finally, lessons could be learned from recommendations made by other experts about making communities see the need for ensuring frequent ANC visits (even when the HFs are closer). This can only be successful if various socio-cultural factors and other supply and demand related barriers to ANC service use are addressed [19,35,46].

## Abbreviations

ANC: Antenatal care; fANC: Focused antenatal care; IPTp: Intermittent preventive treatment during pregnancy; HFs: Health facilities; HWs: Health care workers; MoH: Ministry of health; MoHSW: Ministry of health & social welfare; PMI: US Presidential malaria initiative; SP: Sulphadoxine-pyrimethamine; UNICEF: United Nations Children’s Fund; URT: United Republic of Tanzania; WHO: World Health Organization.

## Competing interests

The authors declare to have no competing interests.

## Authors’ contributions

GMM conceived the study from which the data presented were collected as part of his PhD training. He has, therefore, participated in all stages of the study from after its conception, drafted first and final versions of the manuscript (MS). PB was one of the two principal supervisors of GMM PhD in health sciences study programme; also having reviewed and commented on this MS. All authors read and approved the final manuscript.

## Pre-publication history

The pre-publication history for this paper can be accessed here:

http://www.biomedcentral.com/1472-6963/14/15/prepub
